# Blocking Neurogenic Inflammation for the Treatment of Acute Disorders of the Central Nervous System

**DOI:** 10.1155/2013/578480

**Published:** 2013-05-29

**Authors:** Kate Marie Lewis, Renée Jade Turner, Robert Vink

**Affiliations:** Adelaide Centre for Neuroscience Research, School of Medical Sciences, The University of Adelaide, North Terrace, SA 5005, Australia

## Abstract

Classical inflammation is a well-characterized secondary response to many acute disorders of the central nervous system. However, in recent years, the role of neurogenic inflammation in the pathogenesis of neurological diseases has gained increasing attention, with a particular focus on its effects on modulation of the blood-brain barrier BBB. The neuropeptide substance P has been shown to increase blood-brain barrier permeability following acute injury to the brain and is associated with marked cerebral edema. Its release has also been shown to modulate classical inflammation. Accordingly, blocking substance P NK1 receptors may provide a novel alternative treatment to ameliorate the deleterious effects of neurogenic inflammation in the central nervous system. The purpose of this paper is to provide an overview of the role of substance P and neurogenic inflammation in acute injury to the central nervous system following traumatic brain injury, spinal cord injury, stroke, and meningitis.

## 1. Introduction

Acute disorders of the central nervous system (CNS), including traumatic brain injury (TBI), spinal cord injury (SCI), stroke, and meningitis, account for a significant disease burden worldwide, with CNS injury being the leading cause of death after trauma [1]. These acute neurological conditions affect individuals of all ages and both sexes alike resulting in significant morbidity and mortality. Despite the prevalence of these conditions, current treatments remain limited and largely inadequate. New therapies are urgently required in order to reduce the death and disability associated with these conditions. One feature which is central to each of these conditions is disruption to the blood-brain barrier (BBB)/blood-spinal cord barrier (BSCB) and subsequent development of vasogenic edema. As such, targeting this aspect of the injury cascade is likely to produce beneficial outcomes in each of these conditions. Recent reports on the role of the neuropeptide substance P (SP) and neurogenic inflammation in BBB dysfunction and genesis of cerebral edema following acute brain injury suggest that this pathway provides a novel target for therapeutic intervention. The current paper will provide an overview of the BBB and vasogenic edema, followed by a discussion of the role of SP and neurogenic inflammation in CNS injury.

## 2. Blood-Brain Barrier/Blood-Spinal Cord Barrier

The BBB is a highly selective barrier that serves to protect the fragile brain microenvironment. It is the interface between the blood and the brain, separating the brain parenchyma from the blood within cerebral capillaries, and involves the interactions between endothelial cells, astrocytes, pericytes, and the capillary basement membrane. Within the spinal cord, the blood-spinal cord barrier (BSCB) is similar in function to the BBB [2] and serves to protect the spinal cord by modulating the entry of blood-borne substances. The fundamental structures of the BBB and BSCB are the same although there are some specific differences in the BSCB including glycogen deposits, decreased P-glycoprotein transporters, and decreased expression of tight junctional protein expression [3]. 

The main function of these barriers is to facilitate a constant supply of nutrients, preserve ion homeostasis within the brain/spinal cord microenvironment, and protect against noxious chemicals, variations in blood composition, and the breakdown of concentration gradients. The gate function of the BBB and BSCB is afforded by tight and adherens junctions, comprised of a complex network of transmembrane and cytosolic proteins [4, 5]. Specifically, claudins, occludins, junctional adhesion molecules (JAMs), and zona occludens (ZOs) are the proteins that make up this network. Tight junctions are located on the most apical region of the cleft between cerebral capillary endothelial cells and form a seal to prevent substances from passing between them [6]. Claudins, predominately caludin-5, are involved in the primary makeup or backbone of tight junctions, forming dimers which interact with opposing claudin molecules to form the primary seal of the tight junction [6, 7]. JAM has a single transmembrane segment, which initiates cell-to-cell attachment and is able to mediate permeability through this avenue [7]. Occludin has four transmembrane segments and is present in higher concentrations in endothelial cells of the BBB than in those in systemic capillary endothelial cells. It induces high membrane resistance, which is indicative of low ion permeability [7, 8]. Occludin interacts with the cytoskeleton of BBB/BSCB endothelial cells through ZO1, ZO2, and ZO3 molecules [6, 7]. A further obstacle to prevent the entry of unwanted substances into the brain is provided by the basement membrane of the BBB, which is made up of proteins found within the extracellular matrix including collagens, vitronectin, fibronectin, tenascin, and proteoglycans [9]. These components provide stability to the structure of the blood vessels and a surface upon which cerebral capillary endothelial cells can rest. 

Astrocytes are central to the structure and function of the BBB/BSCB. Their end feet surround 99% of BBB endothelial cells and act to support and enhance the tight junctions between them [7, 10]. Furthermore, astrocytes mediate the connection between neurones and endothelial cells [11], and the gap junctions between astrocytes allow for quick transfer of substances and information [12]. They become activated in response to pathological stimuli, which results in the hypertrophy of the astrocytic processes and overexpression of intermediate filaments, namely, glial fibrillary acidic protein [12].

Pericytes have a stellate appearance and cytoplasmic processes and act as support cells that play an important role in the BBB/BSCB. They cover 22–32% of the capillary cell surfaces [13], and the gap junctions between pericytes and cerebral capillary endothelial cells allow communication to occur [7]. The main function of pericytes is thought to be blood flow regulation, particularly in the precapillary arterioles that supply the brain with blood [14]. The structure of pericytes makes them ideal for this function, as they are contractile and express the smooth muscle actin isoform [13]. Collagen type IV glycosaminoglycans and laminin are also synthesised in pericytes to be used in formation of the basement membrane [13]. They have the ability to regulate endothelial cell proliferation, survival, migration, and differentiation [7]. 

## 3. Edema

Of the secondary injury factors that occur in the setting of CNS injury, edema within the brain or spinal cord is of particular concern given its association with increased mortality and morbidity [15, 16]. Edema is defined as the abnormal accumulation of fluid within the CNS tissue. Klatzo [17] was the first to classify edema into two broad categories based upon the integrity of the BBB: cytotoxic and vasogenic edema. Cytotoxic edema is an intracellular edema that occurs as a result of cellular injury. It is characterized by a shift of water from the extracellular compartment to the intracellular compartment, accompanied by shrinkage of the extracellular space. Cytotoxic edema occurs independently of alterations in the BBB/BSCB and appears to be more prominent in the grey matter [18]. Failure of the Na^+^/K^+^ ATPase in regions of energy failure and subsequent loss of ion homeostasis, leading to influx of water into cells, is central to the development of cytotoxic edema [19, 20]. Conversely, vasogenic edema has been shown to be more prevalent in the white matter [18] and involves the escape of proteins from the vasculature in the setting of BBB/BSCB disruption and injury to cerebral blood vessels. Protein accumulation in the brain/spinal cord extracellular space causes an osmotic increase at the site of injury and the subsequent movement of water down its osmotic gradient. There is a strong correlation between extravasation of proteins into the extracellular space and the development of vasogenic edema [21, 22]. 

The temporal profile of edema pathogenesis after injury varies greatly with injury type and severity [23] and has been extensively studied in order to characterize the period in which anti-inflammatory pharmacological interventions may be effective. In a mouse model of cerebral contusion, permeability of the BBB to large proteins was resolved by approximately 5 hours following injury, whereas smaller molecules of 10 kDa were still able to pass through the BBB for up to 4 days [24]. Similarly, the BSCB may be disrupted for several days following traumatic SCI [25, 26]. Furthermore, in ischemic stroke, it has been shown that edema continues to develop for up to 7 days, with the initial cytotoxic edema being followed by vasogenic edema [27]. Thus, there is substantial opportunity for amelioration of barrier dysfunction and subsequent cerebral edema through manipulation of mediators of BBB/BSCB permeability. Further studies are required to elucidate the exact mechanisms of barrier disruption and subsequent edema pathogenesis to develop targeted therapeutic agents. 

The development of edema is associated with significant mortality and morbidity in the setting of CNS injury. Such outcomes are related to the ability of vasogenic edema to lead to an increase in pressure within the cranium or spinal canal. Given that the skull is rigid structure, any increase in the intracranial contents (blood, brain, and cerebral spinal fluid) must be compensated by a decrease in the volume of the other components. The same is true within the spinal column. Within both the brain and the spinal cord, there is limited capacity for compensation through reductions in blood or cerebrospinal fluid volume to accommodate for an increase in the intracranial volume. This compensation is responsible for the initial plateau in the intracranial pressure/volume curve, which becomes exponential once compensatory mechanisms are exhausted [28]. When such compensatory mechanisms fail, profound increases in intracranial pressure (ICP) or intrathecal pressure (ITP) may result. The sequelae of elevated ICP/ITP include reduced blood flow to CNS tissue, ischemia and infarct extension, deformation and herniation of the brain and spinal cord tissue, and in severe cases, death [18, 29–31]. 

With the mortality of malignant cerebral edema approaching 80% [18], the reduction of cerebral edema and its associated rise in ICP is now widely recognised as an important clinical management target. Current treatments seek to reduce brain swelling and ICP though administration of hyperosmotic agents and barbiturates, induction of hyperventilation or hypothermia, and surgical interventions such as cerebrospinal fluid (CSF) drainage, or in severe cases, decompressive craniectomy [23, 30, 32, 33]. In the case of hemorrhage, evacuation of space occupying lesions like hematomas may be warranted [34]. Clinical signs of edema have been linked with poor functional outcome following SCI [16]. The use of steroids in an attempt to minimize SCI-induced edema and inflammation is common, despite the controversy surrounding their effectiveness and safety [35]. Decompressive surgery is also a current standard treatment following SCI [36]. 

With respect to patient morbidity and mortality, current clinical treatment regimens for acute disorders of the CNS have proven somewhat ineffective, mainly because they do not address the specific mechanisms that are associated with the genesis of edema in cerebral ischemia. Recent studies have identified substance P (SP) release as a feature of acute CNS injury and have delineated a critical role for SP in increased BBB permeability and the development of vasogenic edema. 

## 4. Neurogenic Inflammation

Neurogenic inflammation is a neurally elicited, local inflammatory response characterized by vasodilation, increased vascular permeability, mast cell degranulation, and the release of neuropeptides including SP and calcitonin gene-related peptide (CGRP) [37]. In addition, there are also tissue-specific responses including smooth muscle contraction/relaxation in the bladder and bronchoconstriction in the airways, amongst others [38]. Neurogenic inflammation has been demonstrated in tissue receiving trigeminal innervation and may be stimulated by many agents including prostanoids, leukotrienes, histamine, and serotonin, as well as by changes in the extracellular environment such as decreased pH, increased osmolarity, heat, inflammatory conditions, and tissue (mechanical) injury [39, 40]. The changes in blood vessel size and permeability that occur with neurogenic inflammation lead to edema formation within the tissue [21, 22]. Perhaps the most important factor in this response is SP, having been identified as the most potent initiator of neurogenic inflammation [41, 42].

**Figure 1:**
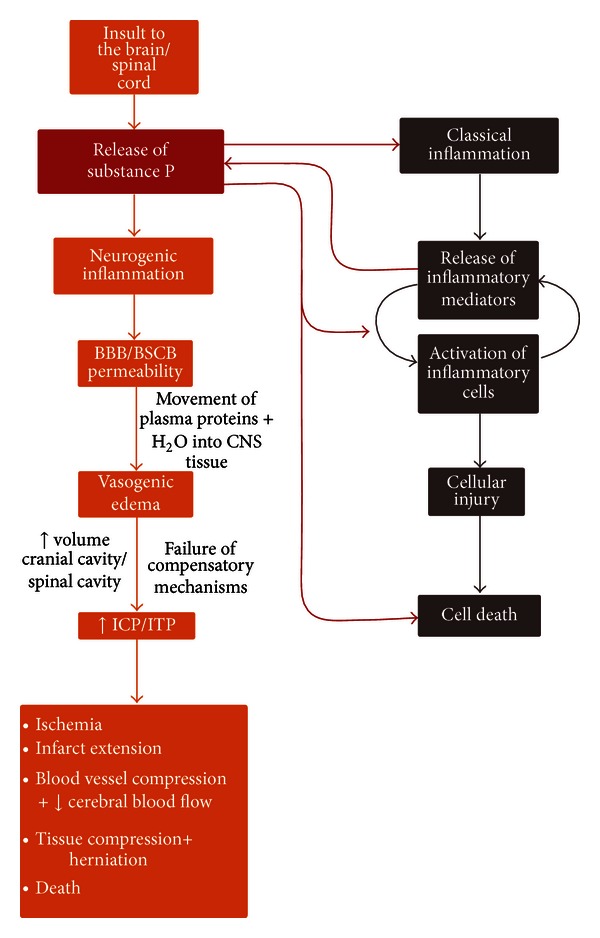
Acute CNS injury leads to the initiation of both neurogenic inflammation and classical inflammation.

Neurogenic inflammatory mediators such as SP and CGRP and their respective receptors are found in abundance in both the rodent and human CNSs, and whilst neurogenic inflammation and classical inflammation are both inflammatory processes, neurogenic inflammation in the brain differs from classical inflammation in that neurogenic inflammation is neurally elicited and results in an increased permeability of the BBB through the release of neuropeptides. In contrast, classical inflammation involves the accumulation and proliferation of microglia, perivascular macrophages, and other inflammatory cells (Figure 1) [43, 44]. These cells subsequently release classical inflammatory mediators like bradykinin, which drive vascular changes [45]. Nevertheless, there is an interaction between the two processes as many of the factors within each cascade may initiate or potentiate the other. For example, the classical inflammatory mediator bradykinin causes release of the neurogenic inflammatory mediator SP, which in turn is well known to cause mast cell degranulation along with bradykinin and nitric oxide release by endothelial cells and thus potentiation of classical inflammation (Figure 1). Inflammation in the brain may play many roles, including the maintenance of tissue homeostasis, although when these processes are unable to be controlled, tissue damage occurs. Thus, this paper focuses on the pharmacological blockade of neurogenic inflammation for the treatment of acute disorders of the CNS. 

There are multiple pathways by which neurogenic inflammation may be initiated. It is well documented, using both animal models and isolated neurons in vitro, that capsaicin, heat, protons, bradykinin, and tryptase are upstream regulators of the intracellular calcium influx, which results in inflammatory neuropeptide release [46–48]. In contrast, it is thought that prostaglandins E_2_ and I_2_, cytokines, interleukin-1, interleukin-6, and tumor necrosis factor do not cause neurotransmitter release themselves, but rather excite sensory neurons and thus lower the threshold for firing and cause augmented release of neuropeptides [48, 49]. 

**Figure 2:**
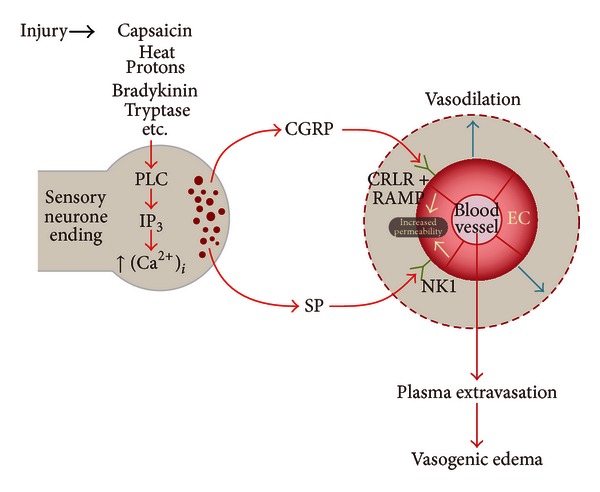
Neurogenic inflammatory initiation of vasogenic edema. PLC—phospholipase C, IP_3_—inositol triphosphate, (Ca^2+^)_*i*_—intracellular calcium ions, CGRP—calcitonin gene-related peptide, SP—substance P, CRLR—calcitonin receptor-like receptor, RAMP—receptor activity modifying protein, NK1—NK1 receptor, EC—endothelial cell.

While neurogenic inflammation has been extensively studied and well documented in peripheral tissues [50, 51], until recently the concept of neurogenic inflammation within the CNS has remained largely unexplored. Given the capacity for neurogenic inflammation to influence vascular permeability and lead to the genesis of edema (Figure 2), it has now been widely investigated for its potential to influence BBB permeability and vasogenic edema within the brain and spinal cord under varying pathological conditions.

### 4.1. Capsaicin

Capsaicin activates transient receptor potential vanilloid-1 (TRPV1) channels on polymodal nociceptive fibers, thus, resulting in the release of neurogenic inflammatory mediators and subsequent vasodilation and increased vascular permeability [52, 53]. Under experimental conditions, capsaicin is commonly used to cause release and/or depletion of neuropeptides [54]. Thus, capsaicin initially elicits a neurogenic inflammatory response, followed by a refractory period in which there is no response to factors that would ordinarily induce neurogenic inflammation. At high doses in young animals, capsaicin may cause permanent damage to the sensory neurons so that neurogenic inflammatory mediators are no longer synthesized, meaning that the neurogenic response is permanently abolished. 

### 4.2. Substance P

SP is an 11 amino acid peptide that is a member of the tachykinin family, so named for their fast-acting properties [55], which also includes neurokinin A (NKA), neurokinin B (NKB), neuropeptide K (NPK), and neuropeptide *γ* (NP*γ*), amongst others. SP is released from both central and peripheral endings of primary afferent neurons where it functions as a neurotransmitter [41, 55]. SP, along with other tachykinins, is produced from the preprotachykinin (PPT) A and B genes. Alternate splicing of the PPTA gene yields the *α*- and *δ*-transcripts giving rise to SP, NKA, NPK, and NK*γ*, whereas the *β*- and *γ*-transcripts only produce SP. The PPTB gene only encodes for NKB. SP synthesis occurs at the cell body ribosomes, where it is then packaged into vesicles and axonally transported to the terminal endings for final enzymatic processing [56]. Precursor proteins are stored in secretory granules along with processing enzymes for posttranslational modifications and release of the active peptide [39, 57]. The biologically active peptide is then stored in large, dense vesicles ready for release. Under normal conditions, substantial amounts of SP are synthesised and stored within neurons [56]. However, activation or damage of these neurons results in the rapid release of SP and other neuropeptides [39].

SP is widely distributed throughout the central and peripheral nervous systems, with *α*-PPTA transcripts more abundant within the brain and *β*-PPTA transcripts more abundant in peripheral tissues. Specifically, in the brain, SP immunoreactivity has been demonstrated in the rhinencephalon, telencephalon, basal ganglia, hippocampus, amygdala, septal areas, diencephalon, hypothalamus, mesencephalon, metencephalon, pons, myelencephalon, and spinal cord. SP has also been found localized within brain endothelial cells and microglia [58–60]. In peripheral tissues, SP and other sensory neuropeptides are distributed throughout the gut, respiratory system, urinary system, immune system, blood, and blood vessels [37]. SP is localized in capsaicin sensitive neurons and is released from central and peripheral endings of primary afferent neurons in response to various noxious stimuli [39]. Of interest is the fact that SP is colocalized with other classical transmitters such as serotonin and glutamate, and other neuropeptides such as CGRP and NKA [56, 59].

Once released, SP may be cleared and inactivated by many different proteolytic enzymes including neutral endopeptidase (NEP) [61, 62] and angiotensin-converting enzyme (ACE) [61, 63, 64], amongst others. Both NEP and ACE catalyse the degradation of the hydrolytic bonds of SP, rendering it inactive without the carboxyl terminus required to bind to its receptor [56]. Specifically, NEP has been shown to degrade SP within the brain [65], spinal cord [66], and peripheral tissues [39], whereas ACE has been shown to degrade SP in plasma, CSF, and brain, in particular the substantia nigra [67].

The biological actions of SP are mediated through its binding at tachykinin NK receptors which are rhodopsin-like membrane structures comprised of 7 transmembrane domains connected by intra- and extracellular loops and coupled to G proteins [68]. To date, 3 mammalian tachykinin receptors have been identified, the NK1, NK2, and NK3 receptors [69]. The tachykinins share a common carboxyl terminal sequence that reflects their common biological action, and, as a result, some cross-reactivity amongst tachykinin receptors exists [70]. Each of the tachykinins may act on all receptor types with varying affinities depending upon receptor availability and neuropeptide concentration. Under normal conditions, SP has a high affinity for the NK1 receptor, NKA for the NK2 receptor, and NKB for the NK3 receptor [38, 71]. The NK1 receptor is a 403 amino acid protein that is highly conserved with only discrete variations amongst species. An NK1 autoreceptor has also been characterized to be involved in the regulation of SP release [72–75]. NK1 receptors are found in their highest levels in the caudate putamen and superior colliculus; however, they are also found in low to moderate levels in the inferior colliculus, olfactory bulb, hypothalamus, cerebral cortex, septum, striatum, mesencephalon, and dorsal horn of the spinal cord [75]. NK1 receptors are expressed by a wide variety of cell types including neurons, astrocytes, oligodendrocytes, endothelial cells, and microglia [76].

SP release is initiated in response to Ca^2+^-dependent depolarisation of neurons, induced by a variety of stimuli including electrical stimulation, pH changes, and ligand binding to their receptors, including capsaicin [37, 57]. Once released, SP has several effects including direct postsynaptic actions as a neurotransmitter, modulatory function at postsynaptic sites, and/or paracrine functions on nonneuronal targets [57]. Transduction of the SP signal then occurs through the action of G proteins associated with the intracellular domain of the NK1 receptor, leading to an elevation in cAMP as a secondary messenger, which through a cascade of events, leads to the regulation of ion channels, enzyme activity, and changes in gene expression [48, 77]. Although normally confined to the cell membrane, the NK1-SP complex is rapidly internalised following SP binding. SP is then removed by endosomal acidification and targeted by the lysosomes for degradation, whilst the NK1 receptor is recycled to the cell membrane [57].

In addition to its role in neurogenic inflammation, SP may induce classical inflammatory reactions through the release of cytokines and recruitment of immune cells. In the skin, SP acts in a dose-dependant fashion to induce mast cell degranulation and histamine and tumour necrosis factor-*α* along with variable release of leukotriene B4 [78, 79]. SP also acts to induce widespread microvascular permeability. Virtually all blood vessels are surrounded by primary sensory nerve fibers that secrete SP, and the cerebral blood vessels are particularly well innervated. Intravenous injection of SP has been shown to increase the permeability of dural blood vessels as evidenced by leakage of horseradish peroxidase in association with widening of junctions between endothelial cells and an increase in the number of cytoplasmic vesicles [80].

In brain endothelial cells, the normal resting level of free Ca^2+^ is 100 nM [81]. SP causes calcium responses in the endothelial cells of the BBB of approximately 1000 nM and hence increase Ca^2+^ levels leading to increased BBB permeability through cell contraction [81, 82]. In conjunction with this, treatment with SP of cerebral capillary endothelial cells cocultured with astrocytes has been shown to decrease the concentration of ZO-1 and claudin-5 tight junctional proteins, resulting in increased permeability of the simulated BBB [83]. 

SP is present in cerebral capillary endothelial cells, and its secretion by these cells can be increased through treatment with high doses of cytokines, including interleukin-1*β* and tumour necrosis factor *α* [60, 84]. This increase in SP released from brain endothelial cells was found to be associated with an increase in the expression of *β*-preprotachykinin mRNA, a precursor for SP, inside the cells [60]. Spantide, a NK1 antagonist, reversed this increase in SP release from endothelial cells and the subsequent increased permeability of the BBB in a dose-dependent fashion [84]. Through the use of electron microscopy, it was shown that the morphological changes associated with SP interactions with endothelial cells were also neutralized [84].

SP has been implicated in the pathogenesis of many neurological diseases, due to its effects on BBB permeability. Thus, NK1 antagonists have been investigated for the treatment of chronic diseases such as Parkinson's [85], depression [86], brain tumours [43, 87, 88], and migraine [89] with variable success. However, this paper focuses primarily on acute disorders of the CNS.

The only NK1 receptor antagonist that is currently available and approved for use clinically is aprepitant. This drug is used as an antiemetic to combat chemotherapy-induced nausea in cancer patients and is generally well tolerated [90]. Thus, NK1 receptor antagonist treatment is an appealing alternative to classical anti-inflammatory drugs, the use of which are often limited by detrimental side effects for the treatment of acute and chronic CNS diseases. 

### 4.3. Calcitonin Gene-Related Peptide

CGRP is a neuropeptide that is commonly colocalized and released with SP, particularly within sensory C fibers that innervate cerebral vasculature [91–94]. CGRP is the most potent endogenous vasodilator [95] and has been shown to increase the diameter of large cerebral arteries and arterioles. This vasodilation has been shown in many species, including the carotid arterial bed of rabbits, piglet arterioles, pial artery of cats, and guinea pigs [96–99]. Furthermore, CGRP infusion in healthy human subjects causes middle meningeal artery dilation [100]. The relaxation of blood vessels by CGRP is mediated by protein kinase C [101]. There are two isoforms of CGRP, CGRP*α* and CGRP*β*, which are encoded by alternate RNA processing of the gene for calcitonin located on chromosome 11 and CGRP*β* [102, 103]. These isoforms differ in only a single amino acid and are functionally similar, although CGRP*α* is the predominate form found in the CNS [104]. CGRP exerts its function through binding at the CGRP receptor, which like the NK1 receptor, has seven transmembrane domains and is coupled to a G protein. The receptor interacts with a single transmembrane receptor activity modifying protein to allow for activation to occur [105, 106]. These receptor complexes are commonly located on neurons, astrocytes, smooth muscle cells, and endothelial cells, particularly those lining dural blood vessels [107–109]. CGRP potentiates the actions of SP [110], which is thought to be through interference with SP breakdown processes [111, 112]. 

## 5. Traumatic Brain Injury

TBI results from physical trauma to the head that consequently causes injury to the brain. It is currently the leading cause of death in individuals under the age of 45 years, with an incidence range of 100–3000 per 100,000 and death rates reported as approximately 18.4 per 100,000 [113–118]. Secondary injury, defined as the persisting alterations to chemicals, cells, and metabolism in the hours and weeks following the primary injury to the brain, is thought to be responsible for substantial cerebral edema and development of neurological deficits [119]. This is of great importance as cerebral edema has previously been shown to be a significant predictor of TBI-induced mortality [15, 18]. 

The majority of TBI cases can be attributed to motor vehicle accidents, motorcycle accidents, bicycle accidents, and pedestrian injuries [120]. Survivors are often are left with debilitating neurological deficits after injury [121, 122], so in addition to the enormous personal burden to victims and their families, the financial impact for the community in terms of hospitalization, treatment, rehabilitation, and specialized care runs into the billions of dollars annually. Despite improvements in motor vehicle safety measures and the marginal decrease in the mortality of trauma-related pathologies [123], TBI still has a significant epidemiological and economic burden on society [124]. 

Outcome following motor vehicle accident-induced trauma is superior in comparison with patients who experience CNS trauma as a result of a fall, likely due to the advanced age generally seen in people who are injured through falling and the younger demographic involved in traffic accidents [125]. Factors that have been implicated in determining the prognosis for patients include age, Glasgow Coma Scale score, arterial hypotension, computed tomography findings, and pupillary reactivity [126]. Childhood populations of trauma victims with evidence of cerebral edema on neuroimaging have shown significantly poorer outcome [15]. Despite this devastating impact, there is currently no approved therapy for the treatment of head trauma, largely because the mechanisms associated with neuronal cell death and the development of cerebral edema are poorly understood. Therefore, recent studies have focused on ameliorating cerebral edema in an attempt to improve recovery following trauma.

TBI results from acceleration/deceleration forces that produce rapid movement of the brain within the skull, or from the head impacting with an object form. The type and severity of the resultant injury are dependent upon the nature of the initiating force, in addition to the site, direction and magnitude of the impact. Injury to the brain following TBI may arise from two different mechanisms, designated as primary and secondary injuries. Primary injury is irreversible, occurring at the time of impact and encompassing the mechanical forces at the time of injury that damage blood vessels, axons, neurons, and glia through shearing, tearing, and stretching [127]. It also includes surface contusions and lacerations, diffuse axonal injury, and hemorrhage. The shearing forces applied to neurons in response to injury cause massive ion fluxes across neuronal membranes, resulting in the widespread loss of membrane potential and the excessive release of neurotransmitters [128]. Such cellular events are part of an evolving sequence of cellular, neurochemical, and metabolic alterations termed as secondary injury, which is initiated by the initial traumatic events and ensues in the hours to days following the initial traumatic event. Secondary injury has profound effects on ion channels, membranes, intracellular biochemical events, and second messenger systems and includes changes in neurotransmitter release, ion homeostasis, blood flow, and cellular bioenergetic state, along with oxidative stress and lactoacidosis [129]. Infiltration of the brain and perilesional area by neutrophils, macrophages, and microglia is also a characteristic of secondary injury and inflammation [130]. Unlike primary injury, such secondary injury is potentially reversible, because its delayed nature provides a therapeutic window for pharmacological intervention. The aim of such therapy is to reduce injury and improve both outcome and survival. However, despite the large number of experimental studies successfully targeting individual injury factors, none have resulted in an effective therapy that can be used clinically. 

*Substance P in Traumatic Brain Injury.* Traumatic brain injury is associated with significant edema formation, proposed by our own group to be mediated by SP and thus neurogenic inflammation. In the human postmortem tissue, SP immunoreactivity is increased following traumatic brain injury [131]. Similarly, perivascular SP immunoreactivity was increased in a rat model of brain trauma, which was closely associated with increased Evans blue leakage into the neuropil, commonly used as an exogenous marker of increased BBB permeability [132]. Animals chronically pretreated with capsaicin, an agent shown to deplete neuropeptides, significantly reduced BBB permeability, cerebral edema, and functional deficits as compared to vehicle-treated controls in a rodent model of diffuse traumatic brain injury [133]. Likewise, NK1 antagonist treatment has been shown to reduce BBB permeability and cerebral edema and to improve functional outcome in this model [132, 134]. Similarly, this treatment has also resulted in amelioration of the proliferative microglial response to diffuse traumatic brain injury [135]. Prevention of SP breakdown with ACE inhibitor treatment also resulted in increased evidence of trauma-induced histological damage and exacerbation of neurological deficits [136]. 

Most of these studies investigating the effects of NK1 antagonist treatment following diffuse traumatic brain injury have been performed in male rats. This is because estrogen may provide additional neuroprotection in females, which could confound experimental results. However, it is important that drugs to treat the complications following traumatic brain injury be effective in both sexes. Recently, an NK1 antagonist treatment has been investigated in an experimental model of trauma in female rats and has been shown to similarly reduce BBB permeability and cerebral edema following injury [137].

Together, these findings make a strong argument for links between elevated perivascular SP and increased BBB permeability leading to cerebral edema formation following both experimental and clinical traumatic brain injuries. Therefore, NK1 antagonist treatment may be beneficial for patients with traumatic brain injury in relieving symptoms of cerebral edema and improving recovery. 

## 6. Traumatic Spinal Cord Injury

Spinal cord injury (SCI) is an insult to the spinal tissue that results in altered motor, sensory, and autonomic functioning. The incidence and mortality estimates for SCI range from 1.3 to 8.3 per 100,000 and 0.3–1.8 per 100,000, respectively, which is approximately 10% of the rates reported for TBI [138–140]. Common mechanisms of SCI are vertebral dislocation and burst fracture injury [141]. Similar to TBI, initial primary injuries including laceration of blood vessels, bone fracture, and axonal injury, are followed by persistent secondary inflammatory processes. Specifically, this commonly includes immune cell accumulation and inflammatory mediatory release, which have been linked to BSCB disruption [142, 143]. The BSCB controls the passage of substances between capillaries and spinal tissue and is disrupted to cause vasogenic edema [144]. This increased permeability of the BSCB may be evident over several segments rostral and caudal from the injury epicenter, particularly following severe spinal cord injury [25, 26]. The importance of this process is illustrated by the established link between edema formation and SCI-induced mortality [16]. 

Nearly 80% of spinal trauma occurs in males, with two peak age groups affected, young adults in their 20s and the elderly over 60 years of age. This bimodal demographic is thought to be associated with traffic accidents and falls, respectively [145]. Brain injury is a common comorbidity for spinal trauma, which is unsurprising as it has many common epidemiological features. The most common site of traumatic spinal cord injury is the cervical level, with decreasing incidence in the lumbar and thoracic regions of the cord [146]. The clinical deficits increase in severity as the SCI occurs at a higher, or more superior, level. 

Spinal cord injury is a highly inflammatory process, resulting in immune cell chemotaxis. In a rodent model of thoracic contusion, inflammatory cytokine release was evident in the spinal cord following injury, which replicates the human condition where a similar pattern of cytokine expression was evident in the CSF, although at a later time point [147]. Additionally, following T9 spinal contusion, neutrophil, macrophages/microglia, and T cells infiltrate the injured spinal cord and remain evident up to 180 days following trauma [148]. 

The promising research on the role of SP in edema development following brain trauma has led researchers to consider that the pathogenesis of secondary injury following spinal cord injury may have similar mechanisms. Moreover, it is thought that this injury type may too respond to manipulation of inflammatory neuropeptides, as it has previously been shown that resolution of BSCB permeability and edema results in improved functional outcome in animal models of SCI [149, 150].   

*Neurogenic Inflammation in Spinal Cord Injury. *Previous studies have shown that SP expression is altered following traumatic SCI in both the human condition and in experimental animal models. In a combined human cohort of both peripheral nerve and SCI patients, increased SP levels in the cerebrospinal fluid were observed in comparison with control patients [151]. Similarly, at both 1 and 2 hours after focal thoracic injury, there was a significant increase in SP found up to 5 mm from the site of injury [152]. In addition, there was a significant increase in brain SP 5 hours after injury [152]. Therefore, the modulation of SP following trauma to the spinal cord may occur throughout the entire CNS. There was also an increase in SP evident following T12 transection of the spinal cord in female cats [153]. In contrast, a weight drop model of trauma in rodents resulted in decreased SP at the site of injury [154]. Furthermore, NK1 receptors have been shown to be significantly increased 1 week after injury using a rat model of thoracic cordotomy [155]. The alterations in both SP and NK1 receptor expression in the spinal cord following trauma suggest that SP may play a part in the pathogenesis of spinal cord injury and its complications. However, further studies are required to determine its exact role. 

There has been limited research on the role of CGRP in traumatic spinal cord injury. It has been shown, following either C4 or T13 hemisection, that primary afferents axons immunostaining for CGRP grow into the area of injury [156, 157]. However, the functional or mechanistic significance of this is yet to be elucidated. Therefore, the evidence for a role of CGRP and possible therapeutic benefit following its manipulation is far less compelling for spinal injury when compared to the results seen for other pathologies. 

## 7. Stroke

Stroke is the third most common cause of disability-adjusted life years and as such is a major health problem worldwide [158]. Specifically, a staggering 15 million people worldwide suffer a stroke each year, of which 10 million either die or are left permanently disabled [159]. The social and economic costs of stroke are consequently enormous. Despite this, there is currently only one approved treatment for use in stroke, that being tissue plasminogen activator within 4.5 h of symptom onset [160]. However, as little as 5–15% of stroke patients are eligible for and receive such treatment. In the case of hemorrhagic stroke, little can be done beyond evacuation of the hemorrhage if surgically accessible. As such, novel therapies that can limit or reverse ischemic injury following stroke are urgently required. 

Stroke is defined as an interruption in the cerebral blood flow of vascular origin that restricts the supply of vital oxygen and substrates for neurons. Stroke can be broadly classified into two types, ischemic and hemorrhagic. Ischemic stroke most frequently involves a thrombus (local origin) or embolus (distant origin) obstructing blood flow, although when blood flow is reestablished, reperfusion injury may occur. This involves the interaction of blood with oxygen-deprived tissue resulting in substantial inflammation and oxidative stress. Hemorrhagic stroke refers to a bleed within the brain. In both instances, cerebral ischemia results, and if blood flow is not rapidly restored, death of cells may result with associated long-term functional deficits [161]. Restoration of blood flow is seen as an urgent priority in reducing the extent of tissue injury and preserving function. However, it is now well accepted that secondary injury processes continue to evolve many hours to days following stroke and also contribute to the size of the infarct [162]. With respect to outcome, hemorrhagic stroke generally has a poorer outcome than ischemic stroke with mortality rates in the order of 37% and 11%, respectively [163]. Hemorrhagic stroke may be classified as either intracerebral hemorrhage (ICH) or subarachnoid hemorrhage (SAH). The rupture of charcot-bouchard microaneurysms on small arterioles commonly leads to ICH, whereas ruptured berry aneurisms within the Circle of Willis are often the cause of SAH [164, 165]. 

Following stroke, the resultant tissue injury and infarction can be considered as being made up of two components, the infarct core and the surrounding penumbral tissue [166]. The infarct core is widely considered to be irreversibly damaged during ischemic stroke, with cell death occurring rapidly within this region. In the penumbral tissue, however, blood flow is less restricted and so there exists an opportunity for neuronal tissue to survive the insult. Nevertheless, cell death may continue to occur here as a result of secondary biochemical and physiological mechanisms that manifest over the hours to days following stroke [162, 166]. Similar to TBI, there are diverse arrays of secondary injury processes that contribute to injury and cell loss following stroke, including excitotoxicity, oxidative stress, inflammation, apoptosis, increased vascular permeability, and cerebral edema, amongst others [167]. Given the delayed nature of secondary injury following stroke, there is an opportunity for pharmacological intervention to limit tissue damage and cell death.

Both SAH and ICH can often result in rapid death, meaning that there is only a small window for therapeutic administration or surgical intervention. Furthermore, given that the mass effect of such hemorrhagic lesions is substantial, the contribution of secondary injury processes to functional impairments is smaller compared with ischemic lesions. In contrast, ischemic stroke has a pattern of injury more comparable to TBI, with increased permeability of the BBB and cerebral edema as common features. Mortality rates increase with time following stroke, demonstrating that even if patients survive the initial insult, the condition may still be fatal due to persistent secondary injury mechanisms such as the development of cerebral edema [168]. The type and severity of edema may be influenced by the duration and severity of ischemia and reperfusion status, amongst other factors, and may also differ between the core and the penumbra of the stroke lesion.

Cerebral edema is a major cause of clinical deterioration within the first 24 h, is the leading cause of death within the first week, and is a predictor of poor outcome following stroke [30]. Clinical studies report that it is maximal between 1 and 3 d following stroke [18], whilst experimental studies report its presence as early as 15 mins after the onset of vascular occlusion [169]. The presence of vasogenic edema is of particular concern, not only because it increases brain volume, but also because in the setting of vascular recanalization, it increases risk of hemorrhagic transformation from damaged blood vessels and excess fluid accumulation [170].

### 7.1. Substance P in Stroke

To date, few groups have investigated SP in cerebral ischemia [171], and only our research group has explored the role of neurogenic inflammation following stroke [172–175]. Our own studies have recently shown that SP is increased following experimental ischemic stroke, indicative of neurogenic inflammation. Specifically, increased SP immunoreactivity was observed within penumbral tissue at 24 h following stroke, being particularly marked in perivascular tissue. Such an increase in SP was confirmed through SP ELISA of the ischemic hemisphere [174]. The increase in SP was associated with marked disruption to the BBB, as evidenced by increased Evan's blue extravasation into the brain parenchyma at 24 h after stroke, thus, supporting previous observations of a delayed opening of the BBB [176]. The increased BBB permeability was observed in the setting of profound cerebral edema, suggesting that the edema had a vasogenic component [174]. Furthermore, profound and persistent functional deficits with respect to motor, sensory, and neurological function were observed [174]. 

A role for SP in clinical stroke has also been documented by Bruno and colleagues [177], suggesting that there may be a role for neurogenic inflammation in this disease pathogenesis. They observed that patients with transient ischemic attack and complete stroke showed elevated serum SP when compared to the control group [177]. Interestingly, individuals with transient ischemic attack showed a greater elevation than complete stroke [177]. 

Early studies reported that hypoxia of the rabbit carotid body increased SP release as a function of the severity of the hypoxic insult [178]. This finding suggested that SP release may be a tissue response to hypoxia/ischemia. Consistent with this, capsaicin pre- or posttreatment was shown to confer protection from neonatal hypoxia-ischemia injury with a reduction in infarct volume and apoptosis, in addition to improved vascular dynamics [179]. 

Given the clear increase in SP that has been documented in both experimental and clinical stroke studies, NK1 tachykinin receptor antagonists have been investigated for their potential utility in reducing BBB dysfunction and vasogenic edema in the setting of ischemic stroke. Yu and colleagues [171] reported a reduction in infarct volume and an improvement in neurological outcome as measured at 24 h poststroke following administration of the NK1 tachykinin receptor antagonist SR-14033. Recently, our group has extended these initial observations and extensively characterized the effect of NK1 tachykinin receptor antagonist treatment in experimental ischemic stroke. Specifically, we have shown that intravenous NK1 antagonist treatment administered 4 hours following stroke resulted in decreased evidence of cerebral edema [174]. Furthermore, when combined with the current standard clot dissolution treatment, tissue plasminogen activator (tPA), NK1 antagonist treatment resulted in equal or better performance in functional outcome tests when compared to NK1 antagonist or tPA alone [175]. 

### 7.2. Substance P in Subarachnoid Hemorrhage

Similar to ischemic stroke, altered SP expression has been reported following SAH. Perivascular SP expression was increased in two models of SAH, injection of autologous blood into the prechiasmatic cistern and following puncture of the middle cerebral artery to cause an endogenous bleed [180]. However, NK1 tachykinin receptor antagonist treatment was unable to ameliorate the raised ICP, cerebral edema, or impaired functional outcome that resulted in either of these models of SAH [180]. A possible reason for this is that the pathogenesis of SAH differs greatly from ischemic stroke, in which NK1 tachykinin receptor antagonists have shown promise. SAH presents less opportunity for therapeutic intervention, due to the mass effect of the bleed, such that therapeutic interventions that act to modulate the permeability of BBB have limited effects. Thus, the functional deficits that result from SAH may be more related to the space occupying blood and damage from its breakdown products rather than to cerebral edema. 

### 7.3. Calcitonin Gene-Related Peptide in Ischemic Stroke

The well-established vasodilatory actions of CGRP have led researchers to postulate that it may play a protective role to promote cerebral blood flow following ischemic stroke. This effect was demonstrated in a rat model of middle cerebral artery reperfusion stroke. Following injury, treatment with CGRP administered at the beginning of reperfusion resulted in a reduction of arterial blood pressure, decreased the infarct volume, and ameliorated the increased BBB permeability subsequently inhibiting cerebral edema formation [181, 182]. 

Along with the vasodilatory actions of CGRP, the mechanism of neuroprotection following ischemic reperfusion stroke may be through modulation of water channels and other elements of the BBB. As such, in two studies using the middle cerebral artery reperfusion model of rodent stroke, CGRP treatment resulted in decreased aquaporin 4 mRNA and protein expression [181, 182]. In conjunction, the reduction in tight junction proteins normally associated with stroke was ameliorated, along with reduced evidence of ultrastructural damage of endothelial cells [181, 182]. Similarly, increased expression of basic fibroblast growth factor has been found following experimental ischemic reperfusion stroke treated with CGRP, which likely acts to improve the structural integrity of the BBB basement membrane [182]. Furthermore, the neuroprotective effects of leptin in a mouse model of middle cerebral artery occlusion and reperfusion injury have been shown to be mediated by CGRP, resulting in increased blood flow and once again reduced infarct volume [183]. Thus, CGRP may be a promising treatment to improve functional outcome following cerebral ischemia through multiple actions on the BBB to reduce the severity of injury. 

### 7.4. Calcitonin Gene-Related Peptide in Subarachnoid Hemorrhage

Akin to ischemic stroke, CGRP is thought to be beneficial following SAH. CGRP has been measured in the cranial venous outflow of 34 patients following SAH and was found to be elevated when compared to the control group, although there was no change in SP levels [184]. In contrast, following subarachnoid hemorrhage, autopsy brain concentrations of CGRP were reduced in comparison with controls in the location of the proximal middle cerebral artery [185]. Therefore, SAH results in modulation of CGRP levels in both the blood and the brain. A possible reason for the differential effects may be that the study in which CGRP was elevated was conducted on patients who had survived their SAH, whilst decreased CGRP was evident following the fatal condition. Thus, the severity of SAH may determine the extent and direction of changes in CGRP. It is postulated that exhaustion of CGRP may be involved in vasospasm, which is most common following severe SAH, and is often a fatal complication. 

CGRP has been tested in both clinical SAH patients and in experimental models of SAH showing protection from abnormal blood vessel contraction. Intravenous administration of human *α*CGRP significantly inhibited vasoconstriction in comparison to that evident prior to infusion in 5 patients [186]. Similarly, when rabbit basilar artery strips were isolated following experimental subarachnoid hemorrhage, responsiveness to in vitro application of CGRP to induce blood vessel relaxation was impaired when compared to those from the control group [101]. This result suggests that increased CGRP levels are required in stroke patients. It is likely that CGRP treatment may hold promise for the prevention of complications associated with subarachnoid hemorrhage. 

Taken together, both animal and clinical studies show that neurogenic inflammation plays an integral role in the pathogenesis of both ischemic stroke and SAH. However, there is a differential effect of inflammatory neuropeptides in these conditions. The role of neurogenic inflammation in ICH has not been widely investigated, although it is likely that, similar to SAH, the edema component of this condition may not contribute as significantly as blood volume to the development of neurological deficits. There is evidence of SP mediation of many deleterious secondary injury mechanisms following ischemic reperfusion injury, including cerebral edema formation. Thus the NK1 receptor is a promising target for pharmacological manipulation to improve patient outcomes. In contrast, SP does not seem to play a significant role in the immediate injury following subarachnoid SAH. CGRP-induced vasodilation may improve blood flow to hypoxic brain tissue during cerebral ischemia and prevent vasospasm following subarachnoid hemorrhage. This indicates that specific neurogenic inflammatory mediators need to be targeted in different ways to optimize treatment following stroke. 

## 8. Bacterial Meningitis

Meningitis is characterized by infection and subsequent acute inflammation of the meninges that cover the outside of the brain. The most common causative infectious agent is bacteria, specifically *Neisseria meningitidis* and *Streptococcus pneumoniae*. There is a marked adult incidence of bacterial meningitis but generally children are most susceptible [187]. Meningitis is associated with CNS symptoms such as neck stiffness, headache, photophobia, phonophobia, altered consciousness, and neurological state, as well as systemic signs of inflammation such as fever, nausea, and vomiting. Additionally, individual bacteria types may be associated with specific features, for example, *Neisseria meningitidis* produces a characteristic rash.

The introduction of vaccinations against specific strains of bacteria has substantially reduced the incidence of this meningitis [188]. Despite this, the availability of antibiotics to combat bacterial infection of the meningitis remains a medical emergency due to the close proximity of inflammation to neurological tissue. This poses a critical threat to brain tissue, not only due to the presence of bacteria, but also the contribution of secondary injury processes. Specifically, inflammatory processes are associated with increased permeability of the BBB and cerebral edema, which worsen the prognosis associated with this disease [6]. Furthermore, cytokine production and leukocyte accumulation are key features in the pathogenesis of bacterial meningitis. 

Currently, anti-inflammatory agents are used in an attempt to combat the symptoms of meningitis, although the dose and duration of treatment are limited by deleterious side effects of the commonly used drugs like the synthetic corticosteroid, dexamethasone. Therefore, alternative therapeutic agents that combat secondary injury and inflammatory processes are attractive targets for investigation. Neurogenic inflammation may be a worthy target given its documented role in BBB permeability and cerebral edema in the setting of acute brain injury and stroke. Specifically, NK1 tachykinin receptor antagonists are able to block neurogenic inflammation by modulating neuropeptide action. In the setting of meningitis, this may prevent deleterious changes in diameter and permeability of cerebral blood vessels and thus leukocyte infiltration and edema formation.

### 8.1. Substance P in Meningitis

In vitro, SP has been shown to increase the production of inflammatory cytokines by astrocytes and microglia when exposed to *Neisseria meningitidis* and *Borrelia burgdorferi* gram-negative bacteria [189]. Similarly, SP treatment of microglia in vitro, which were exposed to the gram-negative *Borrelia burgdorferi* bacteria, results in augmented secretion of prostaglandin E2 [190]. This effect was ameliorated by NK1 tachykinin receptor antagonist treatment and in NK1 knockout cell lines [190]. Furthermore, microglial cells respond to the presence of the gram-positive bacteria *Streptococcus pneumoniae* with upregulation of NK1 receptors by this cell type [191]. These results suggest that NK1 antagonist treatment may act to inhibit many inflammatory processes associated with bacterial meningitis that cause substantial tissue damage and worsen outcome.

Positive results from in vitro studies led to in vivo experiments to determine the effectiveness of NK1 receptor antagonists in experimental mouse models of both gram-positive and gram-negative bacterial meningitis. Intracerebral inoculation of *Neisseria meningitidis* and *Borrelia burgdorferi* into C57BL/6 mice resulted in increased inflammatory cytokine and decreased immunosuppressive cytokine secretion, resulting in a substantially proinflammatory environment [189]. Correspondingly, intracerebral inoculation of female C57BL/6 mice with *Streptococcus pneumoniae* caused a similar pattern of cytokine expression along with gliosis, demyelination, and increased BBB permeability [191]. These features were abolished with both NK1 antagonist treatment and in NK1 knockout mice [189, 191]. Therefore, NK1 antagonist treatment may be able to limit infection-associated inflammation and subsequent edema formation through its ability to inhibit inflammatory cytokine secretion and modulate the permeability of the BBB. The results suggest that in the future, this class of agents could be used as an alternative to classical anti-inflammatory drugs like dexamethasone. However, the effect of NK1 receptor antagonist treatment has only been demonstrated in experimental animal models of meningitis; thus, further investigation into the role of SP in the human condition is required. 

### 8.2. Calcitonin Gene-Related Peptide in Meningitis

The proinflammatory nature of meningitis makes CGRP a likely candidate in the pathogenesis of associated vascular changes, although there has been limited investigation into this area. Nevertheless, patients with acute bacterial meningitis and sepsis have shown evidence of increased CGRP in arterial blood samples [192]. Therefore, the possible role of CGRP in the inflammatory response of bacterial meningitis warrants additional examination. 

## 9. Conclusion

Acute injury to the brain and spinal cord is associated with a number of deleterious secondary injury processes of which altered vascular permeability and tissue swelling are of particular concern. This is further compounded by the lack of effective therapies. However, the inhibition of neurogenic inflammation may provide a novel alternative therapy for the treatment of barrier dysfunction and tissue swelling in the setting of acute CNS injury. Experimental studies of TBI and stroke have shown that blocking the action of SP with an NK1 tachykinin receptor antagonist produces profound reductions in BBB permeability, cerebral edema, and functional deficits. Studies of NK1 tachykinin receptor antagonists in SCI, meningitis, and hemorrhagic stroke are ongoing, but early results suggest that neurogenic inflammation does play a role in these pathologies, albeit a less pronounced role than in TBI and stroke. CGRP may be another worthy target alongside SP with experimental models of both hemorrhagic and ischemic stroke models showing benefits of CGRP treatment. Further investigations on the role of neurogenic inflammation and the neuropeptides SP and CGRP in the barrier dysfunction and tissue swelling that are associated with acute brain and spinal cord injury are ongoing, and given the encouraging results to date, they are certainly warranted.
